# Rehabilitative Kurzzeitpflege (REKUP): Akzeptanz und Praktikabilität eines neuen Versorgungskonzepts

**DOI:** 10.1007/s00391-024-02386-1

**Published:** 2024-12-17

**Authors:** A. Keilhauer, P. Benzinger, S. Diekmann, P. zur Nieden, K. Pahmeier, A. Neumann, J. Wasem, A. Walendzik, T. Hüer, P. Raszke, J. Frankenhauser-Mannuß, N. Specht-Leible, J. M. Bauer

**Affiliations:** 1 Geriatrisches Zentrum am Universitätsklinikum Heidelberg, AGAPLESION Bethanien Krankenhaus Heidelberg, Heidelberg, Deutschland; 2 Institut für Gesundheit und Generationen, Fakultät Gesundheit und Soziales, Hochschule für angewandte Wissenschaften, Kempten, Deutschland; 3 Essener Forschungsinstitut für Medizinmanagement (EsFoMed) GmbH, Essen, Deutschland; 4 Lehrstuhl für Medizinmanagement, Universität Duisburg-Essen, Essen, Deutschland; 5 Unternehmensbereich Versorgungsgestaltung, AOK Baden-Württemberg, Stuttgart, Deutschland

**Keywords:** Poststationäre Versorgung, Rehabilitation, Sektorenübergreifende Zusammenarbeit, Qualitative Forschung, Qualitative Interviews, Posthospitalization care, Rehabilitation, Intersectoral collaboration, Qualitative research, Qualitative interviews

## Abstract

**Hintergrund:**

Rehabilitative Kurzzeitpflege (REKUP) richtet sich an rehabilitationsbedürftige, aber (noch) nicht rehabilitationsfähige geriatrische Patienten nach akutstationärem Aufenthalt mit positiver Rehabilitationsprognose und wurde von Oktober 2020 bis März 2022 an 2 geriatrischen Rehabilitationskliniken erprobt.

**Ziel:**

Qualitative Prozessevaluation hinsichtlich Akzeptanz (Leistungserbringer, -träger), Adoption (Umsetzung zu Projektbeginn), Praktikabilität (Prozesse, Inhalte, Strukturen) und beobachteter Zufriedenheit bei den Patienten.

**Methodik:**

Eine Fokusgruppe und 14 halbstrukturierte Interviews mit insgesamt 18 Mitarbeitenden der Kliniken wie auch des Leistungsträgers wurden aufgezeichnet, transkribiert und qualitativ inhaltsanalytisch ausgewertet.

**Ergebnisse:**

Bei der Befragung der Leistungserbringer (Zuweiser‑/Modellkliniken) als auch des Leistungsträgers zeigten sich eine hohe Akzeptanz sowie eine gute Praktikabilität (Prozesse, Inhalte, Strukturen). Die Umsetzung wurde aufgrund der hohen Akzeptanz sowie der in geriatrischen Rehabilitationskliniken bekannten Leistungsinhalte als gut machbar und die Zufriedenheit der Patienten als sehr hoch bewertet.

**Diskussion:**

Die Akzeptanz von REKUP war hoch, da der Grundgedanke leicht nachvollziehbar war. Dennoch ist eine sorgfältige Patientenauswahl entscheidend. Es besteht die Gefahr, dass REKUP als schnelle Entlassoption genutzt wird, um Kapazitätsprobleme zu umgehen. Der von den Leistungserbringenden wahrgenommene Therapieerfolg konstituiert sich über früh beübende Anwendungen, psychosoziale Angebote und die längere Zeitspanne vor der Rehabilitation: Überforderung wird vermieden und Selbstwirksamkeit gestärkt. Die Aufnahme in den Pflegeheimsektor wird umgangen, was einen positiven psychologischen und motivationalen Effekt für Patienten birgt. Ein Transfer von REKUP in andere geriatrische Rehabilitationskliniken erscheint leicht möglich.

**Zusatzmaterial online:**

Zusätzliche Informationen sind in der Online-Version dieses Artikels (10.1007/s00391-024-02386-1) enthalten.

## Abstract (en)

**Background:**

Rehabilitative short-term care (REKUP) is aimed at geriatric patients in need of rehabilitation but not (yet) capable of rehabilitation after acute inpatient hospitalization with a positive rehabilitation prognosis. It was tested at two geriatric rehabilitation clinics from October 2020 to March 2022.

**Objective:**

Qualitative process evaluation with respect to acceptance (service providers, health insurance), adoption (implementation at the start of the project), practicability (processes, contents, structures) and observed patient satisfaction.

**Methods:**

A focus group and 14 semi-structured interviews with a total of 18 employees of both rehabilitation clinics and a health insurance provider were recorded, transcribed and qualitatively analyzed for content.

**Results:**

The survey of service providers (acute care, model clinics) and the health insurance provider revealed a high level of acceptance and good practicability (processes, contents, structures). Due to the high level of acceptance and the familiar service content in the geriatric rehabilitation clinics, the implementation was feasible and patient satisfaction was rated as very good.

**Conclusion:**

The acceptance of REKUP was high because the basic idea was easy to understand. Nevertheless, a careful patient selection is crucial. There is a risk that REKUP could be used as a quick discharge option to avoid capacity problems. The success of treatment perceived by the service provider is based on early exercises, psychosocial care and a longer period of time before rehabilitation, which avoids excessive demands and strengthens self-efficacy. Admission to the long-term nursing home sector is avoided, which has a positive psychological and motivational effect on patients. A transfer of REKUP to other geriatric rehabilitation clinics seems to be easily possible.

## Hintergrund

Im Anschluss an die Kurzzeitpflege (KZP) nach akutstationärem Aufenthalt wird stationäre Geriatrische Rehabilitation (GR) selten in Anspruch genommen [2, 6]. Die hohe Überleitungsquote in Dauerpflege (DP) lässt Versorgungsdefizite und sektorale Schnittstellenprobleme vermuten [4, 6, 12, 13]. Vor diesem Hintergrund wurde das vom Innovationsfonds des Gemeinsamen Bundesausschuss geförderte Versorgungskonzept der Rehabilitativen Kurzzeitpflege (REKUP) initiiert und von Oktober 2020 bis März 2022 an 2 geriatrischen Rehabilitationskliniken (AGAPLESION Bethanien Krankenhaus Heidelberg und cts Sankt Rochus Kliniken Bad Schönborn) mit jeweils 6 Betten erprobt.

**Abb. 1:**
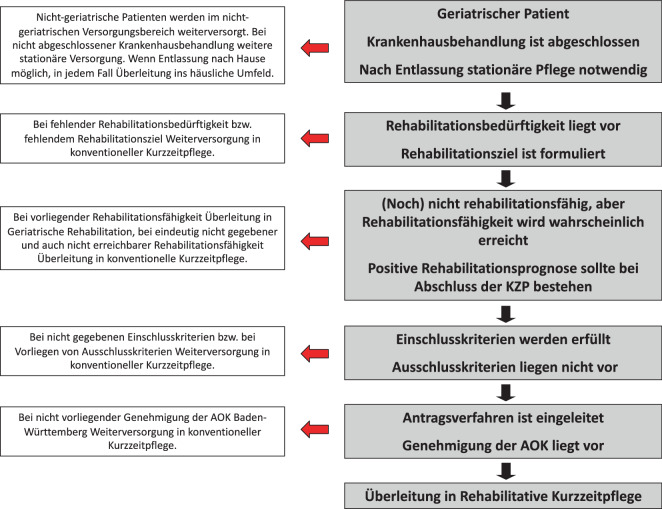
Standardisiertes Screening- und Beurteilungsverfahren der Rehabilitativen Kurzzeitpflege (REKUP)

**Abb. 2:**
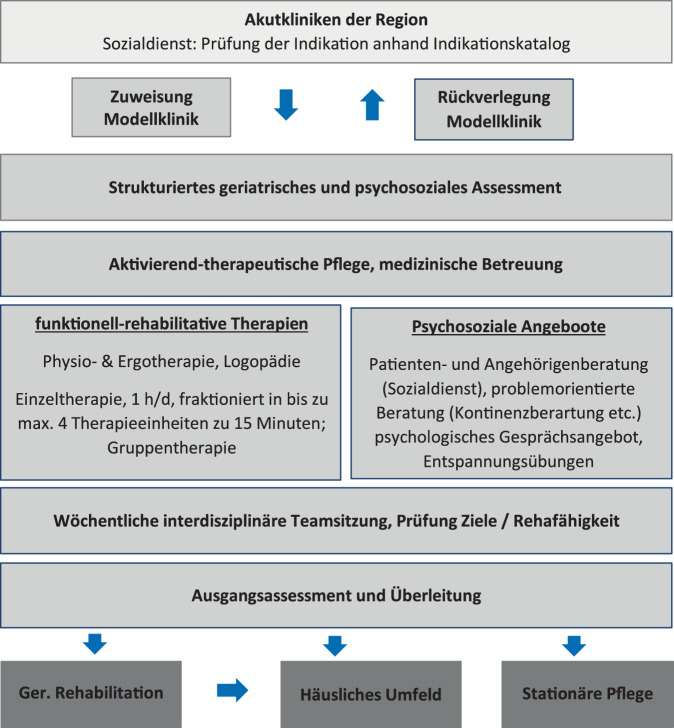
Versorgungskonzept der Rehabilitativen Kurzzeitpflege (REKUP)

Das Angebot der Rehabilitativen Kurzzeitpflege (REKUP) richtete sich an rehabilitationsbedürftige, aber (noch) nicht rehabilitationsfähige geriatrische Patienten nach akutstationärem Aufenthalt mit voraussichtlichem Erreichen von Rehabilitationsfähigkeit, realistischen Rehabilitationszielen und positiver Rehabilitationsprognose gemäß Begutachtungsanleitung „Vorsorge und Rehabilitation“ des GKV-Spitzenverbandes nach § 282 SGB V [11]. Eine Übersicht der Einschluss- und Ausschlusskriterien sowie der Ablauf der Zuweisung kann Abb. 1 und dem ergänzenden Material entnommen werden (Zusatzmaterial online: Supplements 1 und 2). Das REKUP-Programm beinhaltete regelhaft beübende Anwendungen (Physiotherapie, Ergotherapie, Logopädie), aktivierend-therapeutische Pflege, psychosoziale Angebote und medizinische Betreuung durch das Behandlerteam der Rehabilitationskliniken. Entsprechend dem rehabilitativen Ansatz erfolgte bei Aufnahme die Festlegung teilhabeorientierter Ziele. Das Therapieangebot entsprach inhaltlich dem, was üblicherweise in einer GR angeboten wird, wobei die Dauer der Therapieeinheiten aufgrund der verminderten Belastbarkeit der Teilnehmenden reduziert war (maximal 1 h/Tag, unterteilt in bis zu 4 Therapieeinheiten über maximal 15 min Dauer). Die vorgesehene Verweildauer betrug 21 Tage. Eine Übersicht der Interventionsinhalte findet sich in Abb. 2 sowie in den vorausgegangenen Veröffentlichungen zur Wirksamkeit [6, 7] und zu den Kosten [1] von REKUP. Die Rehabilitationsfähigkeit wurde wöchentlich in einer interdisziplinären Teamsitzung geprüft, und REKUP-Teilnehmende (TN) wurden ggf. ohne weiteren Ortswechsel in die GR übergeleitet. Leistungsträger war die AOK Baden-Württemberg (AOK BW) und das Angebot nur deren Versicherten zugänglich. Das REKUP-Programm erprobt ein Versorgungsangebot, welches ergänzend zu der vielerorts nichtverfügbaren mobilen Rehabilitation entwickelt wurde und bestehende Versorgungsstrukturen nutzt.

Ziel der Prozessevaluation war es, REKUP hinsichtlich Akzeptanz (Leistungserbringer, Leistungsträger), Adoption (Umsetzung zu Projektbeginn), Praktikabilität (Prozesse, Inhalte, Strukturen) sowie Zufriedenheit der Patienten zu prüfen.

## Methodik

Die Prozessevaluation erfolgte als qualitatives Studiendesign. Folgende Themenbereiche wurden auf Grundlage einer Fokusgruppe und 14 halbstrukturierter Interviews mit 18 befragten Personen (Leistungserbringer in Zuweiser‑/Modellkliniken sowie Mitarbeitende des Leistungsträgers) qualitativ inhaltsanalytisch ausgewertet:

- *Akzeptanz*: Zufriedenheit mit REKUP bei Mitarbeitenden (MA),
- *Adoption*: förderliche und hinderliche Faktoren der Umsetzung insbesondere zu Projektbeginn,
- *Praktikabilität*:
  - Prozesse: Zugangsverfahren und Zuweisungskriterien (Zuweiserklinik); Aufnahmeverfahren und GR-Überleitung (Modellklinik),
  - Inhalte: Beurteilung der Leistungsinhalte durch verschiedene Bereiche (Pflege, Therapie, Ärzte, Sozialdienst, Administration, Leistungsträger),
  - Strukturen: strukturelle Umsetzungsbarrieren,
- *Zufriedenheit und antizipierte Wirkung*:
  - bei Patienten durch die MA wahrgenommene Zufriedenheit und Einflussfaktoren für Therapieerfolg.

Für genauere Angaben zu den Teilnehmenden an den Interviews und Fokusgruppen verweisen wir auf das ergänzende Material (Zusatzmaterial online: Supplement 3). Die Interviews und die Fokusgruppe fanden zwischen Mai 2022 und August 2022 persönlich oder als Videotelefonat statt. Die Dauer variierte von 15 bis 60 min.

### Interviews und Fokusgruppe

Im Rahmen der 14 Interviews wurden MA aus verschiedenen Klinikbereichen und Verantwortungspositionen befragt: Ärzte, MA der Pflege, Therapie, Verwaltung (Modellkliniken), MA der Kliniksozialdienste (Zuweiser‑/Modellkliniken) und der AOK BW. Vorab wurden berufsgruppenspezifische Interviewleitfäden entsprechend Helfferich [3] erstellt. An REKUP beteiligte MA wurden persönlich von einer Studienmitarbeiterin zur Teilnahme an einem Einzelinterview bzw. zur Teilnahme an einer Fokusgruppe eingeladen. Hierzu wurde ihnen vorab ein Informationsblatt ausgehändigt. Offene Fragen wurden vor den Interviews geklärt und erst nach Vorliegen einer schriftlichen Einverständniserklärung wurde die Audioaufnahme gestartet. Die Interviews wurden per Audioaufnahme aufgezeichnet, transkribiert und pseudonymisiert.

An der Fokusgruppe nahmen teil: Projektleitung der REKUP, Projektmitarbeiterin sowie Pflegedirektorin, Bereichsleitung der KZP und eine Mitarbeiterin des Kliniksozialdienstes (Modellklinik). Ziel war es, unterschiedliche Perspektiven auf die Projektumsetzung zu erhalten. Im Vorfeld wurden zu diskutierende Themen entsprechend den Interviewleitfäden der Einzelinterviews formuliert und die Inhalte anschließend protokolliert.

Die Interviews und die Fokusgruppe wurden qualitativ inhaltsanalytisch und computergestützt durch die Software MAXQDA 2022 (VERBI GmbH, Berlin, Deutschland) sowie strategisch an Kuckarzts inhaltlich strukturierender Inhaltsanalyse [8] orientiert gemeinsam ausgewertet; die Analyse folgte den Gütekriterien qualitativer Forschung [8]. Auf Grundlage der zu erfassenden Themenbereiche wurden vorab deduktive Codes gebildet und während des Auswertungsprozesses auf Grundlage des Datenmaterials induktiv ergänzt. Es wurde ein Codesystem mit Oberkategorien, Codes und Subcodes entwickelt, die zur besseren Trennschärfe mit Code-Memos versehen und mit Ankerbeispielen hinterlegt wurden. Anhand des Codesystems wurde das Datenmaterial von 2 Ratern intersubjektiv nachvollziehbar codiert. Für jeden Code/Subcode wurde ein Text-Retrieval mit allen entsprechend codierten Textstellen sowie daraus abgeleiteten Aussagen der jeweiligen Textstelle erstellt. Die Text-Retrievals wurden per paraphrasierender Zusammenfassung entsprechend den Regeln nach Kuckarzt [8] zusammengefasst und pro Code auf die Kernaussagen reduziert. Durch diese Abstraktion des Datenmaterials konnten generalisierte Aussagen in Bezug auf den jeweiligen Code getroffen werden.

## Ergebnisse

### Akzeptanz

Bei der Befragung der MA zeigte sich eine sehr hohe Akzeptanz von REKUP; keine Berufsgruppe benannte einen Mehraufwand. Sorgen hinsichtlich der Aufnahme von vermeintlich schwerstpflegebedürftigen Patienten erfüllten sich nicht. Die Akzeptanz entstand v. a. durch das Erkennen der Notwendigkeit dieser Versorgungsform zwischen Akutversorgung und Rehabilitation:

„(…) vom Team her war da von Anfang an eine ganz große Akzeptanz, weil (…) diese Versorgungslücke dort zu sehen … von dem her sind wir direkt mit aufgesprungen.“ (LSdK2, 41–43)

Von den MA der AOK BW wurde REKUP ebenfalls als fortsetzungswert erachtet. Allerdings wurde gefordert, REKUP nicht als schnelle Entlassoption in einer prekären nachstationären Versorgungslandschaft zu missbrauchen und verstärkt auf eine medizinische Indikationsprüfung zu achten.

### Adoption (Umsetzung zu Projektbeginn)

Die Umsetzung von REKUP zu Projektbeginn wurde von allen als gelungen bewertet. Als besonders förderlich wurden ein transparenter Informationsfluss sowie strukturierende Unterlagen identifiziert. Eine hohe Akzeptanz im Team wurde als wichtiger Faktor erachtet, ebenso wie eine gute Kooperation mit der AOK BW.

Als hinderlich wurden die Auswirkungen der COVID-19-Pandemie auf den Regelbetrieb, v. a. auf die Kommunikation mit den Zuweiserkliniken, identifiziert. Darüber hinaus wurde ein Informationsdefizit bezüglich des REKUP-Programms bei TN und deren Angehörigen konstatiert.

„(…) und [ich] kann sagen, aufgrund der ganzen wunderbar aufgearbeiteten Unterlagen, die wir bekommen haben, der Leitfaden und der Erklärungen dazu, war es eigentlich ganz unproblematisch, das Ganze hier bei uns mit zu implementieren.“ (LSdK2, Z 35–19)

Gewünscht wurden der Einbezug von mehr Zuweiserkliniken, eine optimierte Patienteninformation, eine angepasste personelle Ausstattung bei möglicher Mehrbelastung durch REKUP als auch eine zentrale REKUP-Ansprechperson in den Modellkliniken.

### Praktikabilität

#### Prozesse

##### Zugangsverfahren und Zuweisungskriterien.

Der Zugang zu REKUP war in den Zuweiserkliniken verankert und oblag der Einschätzung von MA des Sozialdienstes und Ärzten anhand eines Screeningbogens zur Prüfung der Zugangskriterien:

„(…) hat man das [Screeningbogen] (…) ausgefüllt und dann war schon ganz klar ersichtlich, ob das ein REKUP-Patient werden kann.“ (LSdK2, 51–55)

Auch die Antragstellung wurde von allen Befragten als unproblematisch, entsprechend regulärer GR, bewertet. Von Vorteil seien eine Vorabprüfung durch die Modellklinik sowie die sehr gute Zusammenarbeit zwischen Zuweiser‑, Modellklinik und der AOK BW.

Hinsichtlich des Entlassverhaltens der Zuweiserkliniken gaben v. a. MA dieser selbst sowie der AOK BW an, dass REKUP teilweise zu früheren Entlassungen geführt habe. MA der AOK BW wünschten sich eine verstärkte medizinische Indikationsprüfung.

##### Aufnahmeverfahren.

Das Aufnahmeverfahren (Modellkliniken) wurde als reibungslos bewertet; die geringe Fallzahl habe allerdings den Prozess und ein individuelles Vorgehen erleichtert. Die Eingrenzung der Zielgruppe sei angemessen gewesen. In einer der Modellkliniken erfolgten auch bei regulärer GR-Aufnahme eine Prüfung auf Fehlzuweisung und ggf. eine Ummeldung als REKUP-Fall. Die weitere Organisation habe der regulären GR ohne weiteren Mehraufwand entsprochen.

##### GR-Überleitung.

Nach Feststellung der Rehabilitationsfähigkeit im Rahmen der wöchentlichen Teambesprechung schloss sich die GR nahtlos an REKUP an:

„(…) 6 Wochen im gleichen Zimmer, auf der gleichen Station und die brauchten nicht nochmal transportiert werden, das Zimmer wechseln, andere Leute, andere Umgebung. (…) das [war] sicherlich vorteilhafter, als zwischendurch in ein Pflegeheim zu müssen.“ (MaBeQ, 170–176)

Die administrativen Prozesse seien gut handhabbar gewesen, jedoch wurde als wünschenswert beschrieben, den GR-Fall nicht als Neuaufnahme dokumentieren zu müssen.

#### Inhalte

Die Inhalte wurden von den MA der Modellkliniken als sehr gut praktikabel bewertet, da sie sich von den Leistungen einer stationären GR lediglich in der geringeren Dauer der einzelnen Therapieeinheiten unterschieden. Es wurde darauf verwiesen, dass REKUP-Patienten einen teilweise höheren Pflegebedarf aufwiesen. Dieser manifestiere sich in einem erhöhten pflegerischen Aufwand, sodass bei einer zukünftig möglicherweise höheren Zahl an REKUP-Patienten vermehrt auf die Grenzen der Versorgung in einer Rehabilitationsklinik zu achten sei.

Die Notwendigkeit des Angebots im Spannungsfeld zwischen Entlassdruck und verzögerter Rehabilitationsfähigkeit wurde betont, und v. a. die zusätzlichen Behandlungstage durch REKUP im Vorfeld der GR wurden als positiv bewertet. So würden die Gesundheitsentwicklung besser abgesehen und das häusliche Versorgungssetting durch den Sozialdienst entsprechend ausgestaltet werden können:

„(…) ich war ja schon recht früh dann mit den Angehörigen in Kontakt und auch mit dem Patienten. Und dann kann ich das von vornerein begleiten und auch besser organisieren.“ (Ma2SdD, 125–130)

Zudem könne der Stationsarzt bei den hoch vulnerablen Patienten frühzeitig reagieren. Therapeutisch sei REKUP v. a. für TN mit Teilbelastung sinnvoll, da von Beginn an Bewegungsmuster ohne Vollbelastung angebahnt werden könnten:

„(…) wenn (…) Vollbelastung (…) noch nicht da ist, Patienten zu früh kommen, ist einfach der Reha-Erfolg nicht gegeben. Und grad da ist das so, dass man mit einfachen Therapien schon starten kann und dann in der Reha richtig durchstarten kann.“ (Ma1SdD, 99–105)

#### Strukturen

Vor allem die COVID-19-Pandemie belastete im erheblichen Maße die Krankenhausstrukturen – so war v. a. die Kommunikation in die Zuweiserkliniken massiv gestört. Bei z. T. pflegerisch aufwendigen TN wurde eine Verbesserung der personellen Krankenhausausstattung gefordert. Die jetzige Personalausstattung der Rehabilitationskliniken sei nicht ausreichend, um regelhaft solche Patienten zu betreuen. Insbesondere für TN mit Teilbelastung wurde eine flexible REKUP-Verlängerungsoption gewünscht, um die angedachten Erfolge zu erzielen. Zudem wurde von häufigen Fehlzuweisungen noch nicht rehabilitationsfähiger Patienten in die GR und rehabilitativen Versorgungslücken berichtet:

„(…) vielleicht nur noch eine gute Woche, bis der Patient in die Rehafähigkeit gerät (…). Dann ist es schade, wenn wir aufgrund unserer Verweildauern dem Patienten nicht diese Möglichkeit eröffnen können, Rehafähigkeit zu erlangen, (…) wenn man (…) dann ungefördert quasi weiterversorgt und das auch der Endstand bleibt.“ (LSdK2, 136–142)

Hier könne REKUP Abhilfe schaffen, dürfe jedoch nicht als schnelle Versorgungsvariante für ungeeignete Patienten genutzt werden.

### Zufriedenheit und antizipierte Wirkung

#### Wahrgenommene Zufriedenheit

Die beobachtete Zufriedenheit der TN mit REKUP wurde von den MA als hoch eingeschätzt. Ihnen sei bewusst gewesen, dass es sich um ein gutes Angebot handle – auch wenn es eine längere Verweildauer bedeutet habe:

„(…) Hochaltrige, die dann gesagt haben, so lange möchte ich eigentlich nicht von zu Hause weg sein. (…) viele haben dann trotzdem gesagt, ok, (…) den Weg würd’ ich dann trotzdem gehen.“ (MaSdK3, 48–53)

Die TN differenzierten jedoch meist nicht zwischen REKUP, KZP und GR. Der zentrale Punkt habe darin bestanden, nicht ins Pflegeheim zu müssen – aus Sicht der MA hat sich dies positiv auf Motivation und psychisches Wohlbefinden ausgewirkt.

Der nur einmalige Ortswechsel nach Zuweiserklinik habe die Zufriedenheit stark erhöht. Genauso wie das Wegfallen des bei einer KZP zu leistenden finanziellen Eigenanteils bei gleichzeitig sichergestellten Anwendungen und ggf. anschließender GR. Die durch REKUP gewonnenen Behandlungstage ermöglichten stabile therapeutische Betreuung und weniger Überforderung:

„Das ist (…) ein großes Problem, wenn die direkt vom Krankenhaus in die Reha kommen (…) dass sie dann einfach von den Therapien überfordert sind und dann erst nach 2 bis 3 Wochen (…) mit der Reha richtig starten können.“ (Ma1SdD, 105–114)

#### Einflussfaktoren für antizipierte Wirkung

Als grundlegend für die Wirksamkeit von REKUP wurde die geeignete Patientenauswahl erachtet. Zudem sichere REKUP nicht nur körperlich-funktionelle Anwendungen, sondern auch psychosozialer Angebote, was die Wirksamkeit verstärke. Auch die vernetzte Informationsweitergabe im Team wurde als wichtig bewertet.

Die personelle und inhaltliche Kontinuität des Therapieangebots hätten nicht nur Einfluss auf die Akzeptanz, sondern festigten durch die individuelle Begleitung den Therapieerfolg. Der Verbleib in der Rehabilitationsklinik ohne weiteren Ortswechsel sei zentral:

„(…) für Patienten, die (…) delirgefährdet sind (…), dass man die nicht so oft transportieren muss und nicht so oft wechseln muss, ist das sicherlich ein Vorteil.“ (MaBeA, 211–214)

Als maßgeblich für den REKUP-Effekt wurden Akzeptanz, Motivation sowie das subjektive Erleben der TN benannt. So verhinderten kürzere Therapieeinheiten Überforderung und eine rehabilitative Versorgungslücke. Über die durch REKUP verlängerte Zeitspanne hinweg seien Erfolge sichtbarer, wurde Selbstwirksamkeit gefördert und dadurch weitere Fortschritte ermöglicht. Regelmäßige Therapien von Beginn an würden psychischen Problemen vorbeugen:

„(…) wenn die nach 3 Wochen KZP aus einem Pflegeheim kommen und da 3 Wochen nur lagen, ist da sehr viel Frustration einfach, die man einfach umgehen kann, wenn Therapien regelmäßig stattfinden, und dann hat man da auch viel weniger psychische Probleme.“ (Ma1SdD, 289–295)

## Diskussion

Die Prozessevaluation zeigte, dass REKUP bei allen Beteiligten auf sehr gute Akzeptanz stößt, gut umsetzbar ist und eine hohe Zufriedenheit bei den TN beobachtet wurde.

Die Akzeptanz bei den MA der Modellkliniken war hoch, da der Grundgedanke in einer prekären Nachversorgungslandschaft leicht nachvollzogen werden konnte. So kann es im Spannungsfeld zwischen Entlassdruck, Unterversorgung mit KZP-Plätzen und verzögerter Rehabilitationsfähigkeit geriatrischer Patienten zu rehabilitativen Versorgungslücken und vermehrter Pflegebedürftigkeit kommen [2, 5, 9, 10]. Es gab Vorbehalte, dass die Arbeitsbelastung durch den höheren Versorgungsaufwand steigen könne. Eine sorgfältige Patientenauswahl unter Einbezug des Pflegeaufwandes ist deshalb zentral für die Akzeptanz durch die MA.

Für die Zuweiserkliniken mit zunehmendem Entlassdruck [5] war REKUP attraktiv – es wurde über zügigere Entlassungen berichtet. Umso mehr müssen die Indikationen klar angewendet werden, um die Zuweisung ungeeigneter Patienten zu vermeiden und REKUP nicht per Gießkanne anzuwenden, denn nicht jeder geriatrische Patient profitiert gleichermaßen [10].

Entlassdruck bei zu wenigen KZP-Plätzen [5, 9] führt nicht selten zu Fehlzuweisungen noch nicht rehabilitationsfähiger Patienten in die GR. Die in den Interviews genannte Option, den Patienten nach einer Eignungsprüfung durch die Modellkliniken zuerst als REKUP-Patienten aufzunehmen, eröffnet die Möglichkeit, bei solchen Patienten mit fehlender Rehabilitationsfähigkeit diese zunächst im Rahmen von REKUP zu erreichen, bevor die eigentliche GR beginnt. Damit kann deren Rehabilitationspotential besser genutzt werden.

Das REKUP-Programm beinhaltete neben körperlich-funktionellen Anwendungen, aktivierend-therapeutischer Pflege und medizinischer Betreuung auch psychosoziale Angebote, die zentral für den Therapieerfolg waren. So trug beispielsweise die frühe Entlassplanung des Sozialdienstes zur besseren Vorbereitung der häuslichen Versorgungssituation bis zur Entlassung bei. Die durch REKUP gewonnene Zeit vor der GR beugt Überforderung vor und vermeidet eine Therapielücke trotz verlängerter Rekonvaleszenz. Hier scheint REKUP v. a. für TN mit Teilbelastung vorteilhaft, da eine adaptierte Beübung von Anfang an möglich ist.

Als ein der GR vorausgehendes Angebot greift REKUP auf vorhandene Strukturen, Behandlerteams und -verfahren zurück. Vor diesem Hintergrund scheint ein Transfer in andere geriatrische Rehabilitationskliniken unter der Voraussetzung leicht möglich, dass in den entlassenden Akutkliniken der Rehabilitationsbedarf trotz fehlender Rehabilitationsfähigkeit erkannt wird, Kapazitäten in Rehabilitationskliniken vorhanden sind sowie die Finanzierung gesichert ist.

## Resümee

Die Prozessevaluation zeigte eine sehr hohe Akzeptanz von REKUP bei MA der Modellklinik wie auch der AOK BW. Es wurde jedoch darauf hingewiesen, dass bei höherer Fallzahl und ggf. erhöhtem Pflegeaufwand vermehrt auf die Grenzen der Versorgungsmöglichkeiten in einer Rehabilitationsklinik zu achten ist. Maßgeblich für die erfolgreiche Umsetzung zu Projektbeginn war die Akzeptanz unter den MA, die sich v. a. aus dem Erkennen der Notwenigkeit des Versorgungskonzepts ergab. Prozesse, Inhalte und Strukturen waren entsprechend regulärer GR gut etabliert und deshalb einfach umsetzbar. Das Angebot REKUP stellt für eine ausgewählte Gruppe geriatrischer Patienten eine vielversprechende Option dar, die trotz einer positiven Rehabilitationsprognose noch nicht über die erforderliche Belastbarkeit für eine GR verfügen und keinen Zugang zu einer mobilen Rehabilitation in einer Kurzzeitpflegeeinrichtung haben. Diese Patienten sind derzeit unterversorgt und werden nur selten in einem gebesserten Zustand von dort einer GR zugeführt [6, 7]. Das Versorgungskonzept REKUP darf jedoch nicht als schnelle Versorgungslösung für ungeeignete Patienten missbraucht werden.

Die beobachtete Zufriedenheit der TN mit REKUP erscheint ebenfalls sehr hoch. Dies kann erklärt werden durch die Vermeidung eines weiteren Ortswechsels, den Wegfall des bei einer KZP zu leistenden finanziellen Eigenanteils und gesicherte Therapien bei ggf. anschließender GR mit potenziell besserem Gesamtergebnis. Auch die durch REKUP gewonnene Behandlungszeit vor der GR wurde positiv bewertet, da dies Überforderung vermeide und bessere Therapieerfolge ermögliche. Zudem wird durch REKUP der Pflegeheimsektor nicht betreten – mit positiven psychologischen und motivationalen Effekten für die TN.

## Fazit für die Praxis

- Das Versorgungskonzept REKUP greift auf vorhandene Strukturen, Behandlerteams und -verfahren zurück und war daher leicht umsetzbar.
- Überleitung in die GR ohne Ortswechsel, Wegfall des bei einer KZP zu leistenden finanziellen Eigenanteils und gesicherte Therapie wurden als besonders positiv bewertet.
- Eine sorgfältige Patientenauswahl ist zentral für die Akzeptanz unter den MA.
- Der Therapieerfolg konstituiert sich u. a. durch frühe Anwendungen, psychosoziale Betreuung als auch die zusätzlichen Behandlungstage vor der GR.
- Mit REKUP wird die KZP wird nicht zum Einfalltor für DP mit positiven psychologischen und motivationalen Effekten.

## Supplementary Information

Supplement 1 Screeningbogen

Supplement 2 Leitfaden Screening

Supplement 3 Interviews

## Data Availability

Die Daten können aus Datenschutzgründen nicht zur Verfügung gestellt werden.
