# Supplementary material for: Rehabilitative short-term care (REKUP): acceptance and practicability of a new care concept
Source: Z Gerontol Geriatr. 2024 Dec 17;58(4):282–8. [Article in German] doi: 10.1007/s00391-024-02386-1 (PMC12238107; doi:10.1007/s00391-024-02386-1)
Supplement: Supplementary file 1 — Supplement 1 Screeningbogen [file 391_2024_2386_MOESM1_ESM.pdf]

REKUP – Rehabilitative Kurzzeitpflege

Aufnahmeverfahren / Screening

Patienten-ID:____________________

Eingangsvoraussetzungen

Geriatrischer Patient [1]	Ja □	Nein □
Krankenhausbehandlung abgeschlossen [2]	Ja □	Nein □
nach Entlassung ist stationäre Pflege notwendig [3]	Ja □	Nein □

Gesundheitsbezogene Punkte

Rehabilitationsbedürftigkeit liegt vor [4]	Ja □	Nein □
Rehabilitationsziel ist formuliert [5]	Ja □	Nein □

____

(Noch) nicht rehabilitationsfähig, aber Rehabilitationsfähigkeit wird wahrscheinlich erreicht [6]	Ja □	Nein □
Positive Rehabilitationsprognose sollte bei Abschluss der KZP bestehen [7]	Ja □	Nein □
Einschlusskriterien werden erfüllt [8]	Ja □	Nein □
Ausschlusskriterien wurden ausgeschlossen [9]	Ja □	Nein □
