# Supplementary material for: Rehabilitative short-term care (REKUP): acceptance and practicability of a new care concept
Source: Z Gerontol Geriatr. 2024 Dec 17;58(4):282–8. [Article in German] doi: 10.1007/s00391-024-02386-1 (PMC12238107; doi:10.1007/s00391-024-02386-1)
Supplement: Supplementary file 2 — Supplement 2 Leitfaden Screening [file 391_2024_2386_MOESM2_ESM.pdf]

Rehabilitative Kurzzeitpflege (Modellprojekt REKUP)

AOK Baden-Württemberg

AGAPLESION Bethanien Krankenhaus Heidelberg

cts Sankt Rochus Kliniken Bad Schönborn

Leitfaden zur Identifikation geeigneter Patienten / Indikationsstellung

Die Identifikation geeigneter Patienten für das neue stationäre Versorgungsangebot der rehabilitativen Kurzzeitpflege sowie die Indikationsstellung zur Überleitung in rehabilitative Kurzzeitpflege erfolgt über ein standardisiertes Verfahren (Screening), bei dem geltende Begutachtungsanleitungen und Abgrenzungskriterien zur geriatrischen Rehabilitation Anwendung finden (siehe Anhang). Die nachfolgenden Definitionen und Abgrenzungskriterien sind dabei zu beachten.

ACHTUNG

Partner des Versorgungsvertrags zur Finanzierung der rehabilitativen Kurzzeitpflege ist während der Modellphase die AOK Baden-Württemberg. In rehabilitative Kurzzeitpflege können deshalb nur geriatrische Patienten übergeleitet werden, die bei der AOK Baden-Württemberg krankenversichert sind.

1 Geriatrischer Patient

Zielgruppe der rehabilitativen Kurzzeitpflege sind geriatrische Patienten. Wesentliche Merkmale geriatrischer Patienten sind:

- die geriatrietypische Multimorbidität und ein höheres Lebensalter (> 70 Jahre)
- durch akute und chronische Erkrankungen verursachte Einschränkungen der Mobilität, der Kognition und des Affekts, der Sensorik und der Kommunikation, der Selbstversorgung sowie evtl. drohende oder bereits bestehende Pflegebedürftigkeit
- ein geriatrisches Risikoprofil; z. B. Sturzgefährdung, Polypharmazie, Ernährungsprobleme, Muskelabbau und Kraftverlust (Sarkopenie), wiederholte Klinikaufenthalte und Komplikationen
- eine allgemein erhöhte Vulnerabilität bei eingeschränkten Reservekapazitäten

Keilhauer A. et al. Rehabilitative Kurzzeitpflege (REKUP): Akzeptanz und Praktikabilität eines neuen Versorgungskonzepts für geriatrische Patienten im rehabilitativen Setting – eine qualitative Analyse

2 Krankenhausbehandlung ist abgeschlossen

Krankenhausbehandlungsbedürftigkeit liegt nicht mehr vor, wenn:

- die vitalen / vegetativen Parameter (Herzkreislauf, Blutdruck, Atmung, Temperatur) stabil sind
- die Diagnostik und die Akuttherapie der zur Aufnahme führenden Erkrankung, der Begleiterkrankungen sowie evtl. Komplikationen abgeschlossen ist
- eine adäquate Weiterversorgung nicht mehr der besonderen Mittel der Krankenhausbehandlung bedarf, sondern ambulant gewährleistet ist

Bei Patienten, die in rehabilitative Kurzzeitpflege übergeleitet werden, muss die Krankenhausbehandlung abgeschlossen sein. Sie stehen zur Entlassung an, und weitere Krankenhausbehandlung ist nicht mehr erforderlich.

Diagnostische Maßnahmen oder die Durchführung von i.v.-Therapien sind in der rehabilitativen Kurzzeitpflege (entsprechend der Situation in konventioneller Kurzzeitpflege) nicht möglich. Eine regelmäßige ärztliche Überwachung gesundheitlicher Risiken ist gewährleistet.

3 Nach Entlassung aus der Akutklinik ist stationäre Pflege notwendig

Patienten, bei denen nach Abschluss der Behandlung im Akutkrankenhaus eine Entlassung zurück ins häusliche Umfeld möglich und gesichert ist, werden aus der Akutklinik ins häusliche Umfeld übergeleitet.

Bei Patienten, die in rehabilitative Kurzzeitpflege übergeleitet werden, ist eine Entlassung zurück ins häusliche Umfeld nicht möglich, da die pflegerische Versorgung im häuslichen Umfeld nicht gesichert bzw. zum Zeitpunkt der Entlassung aus dem Krankenhaus noch nicht gesichert oder unklar ist.

Bedarf an stationärer Pflege besteht nicht nur für einen absehbaren Zeitraum von wenigen Tagen. Der Bedarf an stationärer Pflege und damit auch an Kurzzeitpflege ist primär durch die vorliegenden körperlichen Einschränkungen verursacht, und nicht durch das Fehlen einer häuslichen Pflegeperson oder einer noch unzureichenden Ausstattung bzw. Anpassung des häuslichen Umfelds.

4 Rehabilitationsbedürftigkeit liegt vor

Rehabilitationsbedürftigkeit besteht beim Vorliegen von nicht nur vorübergehenden alltagsrelevanten Beeinträchtigungen der Aktivitäten, der Selbstversorgung und der Teilhabe aufgrund einer körperlichen, geistigen oder seelischen Schädigung. Zu den Beeinträchtigungen der Teilhabe gehört auch der Zustand der Pflegebedürftigkeit.

Die vorliegenden Beeinträchtigungen der Aktivitäten und der Selbstversorgung bilden sich im Barthel-Index ab.

Über die kurative Versorgung im Akutkrankenhaus hinaus ist ein mehrdimensionaler, interdisziplinärer Ansatz der medizinischen Rehabilitation erforderlich. Es besteht Bedarf für aktivierend-

therapeutische Pflege und spezielle therapeutische Leistungen wie Physiotherapie, medizinische Trainingstherapie, Ergotherapie, Logopädie oder psychosoziale Unterstützung.

Nach Feststellung einer Rehabilitationsbedürftigkeit sind möglichst frühzeitig rehabilitative Maßnahmen einzuleiten, um Besserungschancen zeitnah zu mobilisieren und weiterem Abbau vorzubeugen.

In Abgrenzung zur indikationsspezifischen Rehabilitation sind bei der geriatrischen Rehabilitation mehr als eine Erkrankung bzw. Krankheitsfolge zu berücksichtigen. Der Rehabilitationsbedarf der Zielgruppe rehabilitativer Kurzzeitpflege ist primär somatisch begründet.

5 Rehabilitationsziel ist formuliert

Übergeordnete Rehabilitationsziele sind der Erhalt, die Verbesserung und ein Wiedergewinn größtmöglicher Selbstständigkeit, Selbstbestimmung und Teilhabe; insbesondere auch Vermeidung oder Verminderung von Pflegebedürftigkeit.

Konkrete Rehabilitationsziele können sein:

- Verbesserung der Sitzstabilität
- Verbesserung der Rollstuhlfähigkeit
- Verbesserung von Transfer, Stehen und Gehen
- Verbesserung des Toilettengangs
- Gehfähigkeit über mehrere Treppenstufen
- Verbesserung der Kommunikation
- Verbesserung der Tagesstrukturierung (z. B. zur Gewährleistung zeitgerechter Medikamenten- und Nahrungsaufnahme)
- Reduzierung des Bedarfs an Fremdhilfe

Vor Überleitung in rehabilitative Kurzzeitpflege muss ein konkretes individuelles, alltagsrelevantes und realistisches Rehabilitationsziel für den Patienten formuliert werden.

6 (Noch) nicht rehabilitationsfähig, aber Rehabilitationsfähigkeit wird wahrscheinlich erreicht

Rehabilitationsfähigkeit ist die ausreichende körperliche und geistige Belastbarkeit für die mehrmals tägliche aktive Teilnahme an rehabilitativen Maßnahmen. Der Zugang zur geriatrischen Rehabilitation ist dabei niedrigschwelliger ausgelegt als bei der indikationsspezifischen Rehabilitation.

Geriatrische Rehabilitationsfähigkeit liegt vor, wenn:

1. die vitalen / vegetativen Parameter (Herzkreislauf, Blutdruck, Atmung, Temperatur) stabil sind
2. die klinische Diagnostik abgeschlossen ist

3. die medizinische, besonders medikamentöse Therapie weitgehend festgelegt ist
4. keine Notwendigkeit zur dauernden Beaufsichtigung besteht (Hinlauftendenz, Eigen- und Fremdgefährdung)
5. die bestehenden Begleiterkrankungen und Komplikationen vom Fachpersonal der Rehabilitation adäquat behandelt werden können
6. bestehende Begleiterkrankungen und Komplikationen die aktive Teilnahme an einer geriatrischen Rehabilitation nicht verhindern
7. die Stabilität des Kreislaufs und die allgemeine Belastbarkeit des Patienten mehrmals täglich aktive rehabilitative Maßnahmen von mindestens 15 Minuten Dauer im Sitzen erlauben

Bei vorliegender geriatrischer Rehabilitationsfähigkeit kann bei geriatrischen Patienten eine stationäre oder ambulante geriatrische Rehabilitation indiziert sein. Patienten, die in rehabilitative Kurzzeitpflege übergeleitet werden, erfüllen jedoch die oben genannten Kriterien nicht vollständig. Sie sind (noch) nicht rehabilitationsfähig.

Die Punkte 1-5 müssen erfüllt sein, sonst ist eine Übernahme in rehabilitative Kurzzeitpflege nicht möglich. Die Punkte 6 und 7 dürfen bei Personen in rehabilitativer Kurzzeitpflege jedoch (noch) nicht erfüllt sein. Durch vorliegende Erkrankungen, Begleiterkrankungen und Komplikationen ist die aktive Teilnahme an rehabilitativen Maßnahmen nicht oder nur in zu geringem Maße gegeben. So erlaubt die gegenüber rehabilitationsfähigen Patienten verminderte Belastbarkeit lediglich nur eine passive Teilnahme oder nur geringere Umfänge einer aktiven Teilnahme an rehabilitativen Maßnahmen als unter Punkt 7 vorgegeben. Aus diesem Grund ist die Zielgruppe der rehabilitativen Kurzzeitpflege noch nicht hinreichend rehabilitationsfähig. Aufgrund der gravierenderen funktionellen Einschränkungen ist der Barthel-Index bei Zuweisungen zur rehabilitativen Kurzzeitpflege niedriger als bei Zuweisungen zur geriatrischen Rehabilitation. Der Punktwert wird in der Regel unter 35 liegen. Gleichzeitig ist bei den Patienten wahrscheinlich, dass durch die Inanspruchnahme einer rehabilitativen Kurzzeitpflege Rehabilitationsfähigkeit erreicht werden kann.

Beispiele sind Patienten mit nicht-belastungsstabil versorgten Frakturen, Patienten mit erheblichem Muskel- und Kraftabbau im Rahmen eines langwierigen und komplikationsreichen Krankheitsverlaufs oder Patienten, bei denen im Rahmen des Klinikaufenthalts ein Delir auftrat (siehe auch Patienten-Vignetten).

7 Positive Rehabilitationsprognose sollte bei Abschluss der KZP bestehen

Die Rehabilitationsprognose ist eine medizinisch begründete Wahrscheinlichkeitsaussage für den Erfolg der Leistung zur medizinischen Rehabilitation.

Bei positiver Rehabilitationsprognose ist die Erreichbarkeit des Rehabilitationsziels durch geeignete Maßnahmen in einem notwendigen Zeitraum wahrscheinlich, unter Berücksichtigung des bisherigen Verlaufs und vor dem Hintergrund relevanter Kontextfaktoren.

Bei der Prognosestellung ist zu berücksichtigen, dass mit zunehmendem Alter aufgrund reduzierter Reserven sowohl die Chancen einer kompletten Rückbildung von Schädigungen auf Körperebene (Restitution) als auch deren Ausgleichsmöglichkeiten über andere Körperfunktionen (Kompensation)

abnehmen. Für den Wiedergewinn von Aktivitäten und Teilhabe kommt daher im Alter umso mehr der Ausgestaltung von umwelt- und personenbezogenen Kontextfaktoren (Adaptation) Bedeutung zu.

Eine positive Rehabilitationsprognose für die geriatrische Rehabilitation ist anzunehmen, wenn mindestens eines der nachfolgend genannten Kriterien zutrifft:

- Beseitigung oder Verminderung der alltagsrelevanten Beeinträchtigungen durch Verbesserung der Selbsthilfefähigkeit sind erreichbar
- Strategien zur Alltagsbewältigung (Kompensation) sind mit Aussicht auf nachhaltigen Erfolg anzuwenden (trainierbar)
- Anpassungsmöglichkeiten (Adaptation) sind vorhanden und nutzbar

Bei Überleitung in rehabilitative Kurzzeitpflege liegt eine positive, evtl. eine noch unklare Rehabilitationsprognose vor.

8 Einschlusskriterien werden erfüllt

Eine Überleitung in rehabilitative Kurzzeitpflege ist möglich bei multimorbiden geriatrischen Patienten mit internistischen, chirurgischen, orthopädischen, neurologischen und seltener gerontopsychiatrischen Erkrankungen, die die zuvor genannten Kriterien (Punkte 1-7) erfüllen.

Nach der Hauptbehandlungsdiagnose in der Akutklinik sowie nach Pflegestatus werden die Patienten verschiedenen Fallgruppen zugeordnet (diese Zuordnung ist nicht Aufgabe des Screenings).

In den Patienten-Vignetten sind einzelne Zielgruppen der rehabilitativen Kurzzeitpflege beispielhaft nochmals beschrieben.

9 Ausschlusskriterien liegen nicht vor

Die Versorgung von Patienten mit nachfolgend gelisteten Erkrankungen, Komplikationen oder versorgungsrelevanten Einschränkungen ist in rehabilitativer Kurzzeitpflege nicht zu gewährleisten. Die nachfolgenden Merkmale stellen deshalb Ausschlusskriterien dar:

- Patienten in terminalen Erkrankungsphasen
- fehlende Aussicht auf die Herstellung einer ausreichenden Belastbarkeit für eine aktive Teilnahme an einer Rehabilitationsmaßnahme
- lebensbedrohliche Akuterkrankung
- vorliegender Pflegegrad >3
- fortgeschrittene Demenzerkrankung
- Suchterkrankung
- keine ausreichende Kommunikationsfähigkeit
- ständige Beaufsichtigung erforderlich bei Hinlauftendenz, Eigen- oder Fremdgefährdung
- aufwändige Wundversorgung
- dialysepflichtiger Patient

- isolationspflichtiger Patient
- Tracheostoma
- kontinuierlicher O_2-Bedarf

Der Punktwert des Barthel-Index wird bei Personen, die in rehabilitative Kurzzeitpflege übergeleitet werden, in der Regel unter 35 liegen. Ein höherer Punktwert im Barthel-Index ist jedoch für sich allein kein Ausschlusskriterium.

10 Antragsverfahren eingeleitet

Der Kliniksozialdienst bzw. der zuständige Arzt der Akutklinik führen im Rahmen des Entlassmanagements das Screening durch. Bei positivem Screening füllen Sozialdienst und Stationsarzt das Standard AHB-Formular für den betreffenden Patienten / Versicherten aus und schicken diesen mit dem Screeningbogen an das zuständige CC-Reha der AOK Baden-Württemberg.

Anträge aus Akutkliniken in Heidelberg / Mannheim / Rhein-Neckar-Kreis an:

AOK-Bezirksdirektion Rhein-Neckar-Odenwald

CompetenceCenter Rehabilitation

Renzstr. 11-13 - 68161 Mannheim

Telefon: 0621 176 - 8867

Fax: 0621 176 - 918867

Anträge aus Akutkliniken in Karlsruhe / Bruchsal / Bretten an:

AOK-Bezirksdirektion Mittlerer Oberrhein

CompetenceCenter Rehabilitation

Kriegsstr. 41 - 76133 Karlsruhe

Telefon: 0721 3711 - 620

Fax: 0721 3711 - 91620

Gleichzeitig klärt der Sozialdienst die Aufnahme des Patienten zur rehabilitativen Kurzzeitpflege in einer der beiden Modelleinrichtungen:

AGAPLESION Bethanien Krankenhaus Heidelberg

cts Sankt Rochus Kliniken Bad Schönborn

11 Genehmigung der AOK liegt vor

Sobald die Genehmigung der AOK vorliegt, kann der Patient nach Rücksprache mit der aufnehmenden Klinik in rehabilitative Kurzzeitpflege übergeleitet werden. Für den Transport muss eine Verordnung organisiert werden. Dies ist wichtig für die Übernahme der Fahrtkosten durch die Krankenkasse.

Bei fehlender Bewilligung erfolgt alternativ eine Entlassung in konventionelle Kurzzeitpflege.

Geltende Richtlinien / Abgrenzungskriterien

Begutachtungsanleitung Vorsorge und Rehabilitation. Richtlinie des GKV-Spitzenverbandes nach §282 SGB V vom 2.7.2018.
