# Supplementary material for: Rehabilitative short-term care (REKUP): acceptance and practicability of a new care concept
Source: Z Gerontol Geriatr. 2024 Dec 17;58(4):282–8. [Article in German] doi: 10.1007/s00391-024-02386-1 (PMC12238107; doi:10.1007/s00391-024-02386-1)
Supplement: Supplementary file 3 — Supplement 3 Interviews [file 391_2024_2386_MOESM3_ESM.pdf]

Übersicht der geführten Interviews

Interview/Bereich	Datum	Teilnehmer*innen/Bereich	Einrichtung	Pseudonym
**FOKUSGRUPPE**				
Fokusgruppe 1	09.05.2022	Pflegedirektor*in Modellklinik; Bereichsleitung Geriatrie/ Kurzzeitpflege Modellklinik 2, Mitarbeiter*in Sozialdienst Modellklinik 2, Projektleitung REKUP, Wissenschaftliche*r Mitarbeiter*in REKUP	Modellklinik 1<br>Modellklinik 2	PflDD, BlD, Ma3SdD, PL, PMa
**EINZELINTERVIEW**				
Pflege	22.06.2022	Bereichsleitung Pflege geriatrische Rehabilitation	Modellklinik 1	MaPfA
Sozialdienst	27.06.2022	Leitung Sozialdienst	Akutklinik 1	MaSdK1
Arzt	27.06.2022	Chefärzt*in	Modellklinik 1	ChaA
Physiotherapie	30.06.2022	Mitarbeiter*in Physiotherapie geriatrische Rehabilitation	Modellklinik 1	MaPhA
Verwaltung	06.07.2022	Mitarbeiter*in Belegungsmanagement	Modellklinik 1	MaBeA
AOK	07.07.2022	Leitung Competence Center Rehabilitation	CC Reha AOK	Lcc
Arzt	15.07.2022	OberÄrzt*in	Modellklinik 2	OaD
Sozialdienst	27.07.2022	Mitarbeiter*in Sozialdienst	Modellklinik 2	Ma1SdD
Sozialdienst	27.07.2022	Mitarbeiter*in Sozialdienst	Modellklinik 2	Ma2SdD
Sozialdienst	28.07.2022	Leitung Sozialdienst	Akutklinik 2	LSdK2
Sozialdienst	29.07.2022	Mitarbeiter*in Sozialdienst	Akutklinik 3	MaSdK3
Sozialdienst	05.08.2022	Mitarbeiter*in Sozialdienst Rehaklinik	Modellklinik 1	MaSdA
**DOPPELINTERVIEW**				
Pflege	15.07.2022	Mitarbeiter*in Pflege geriatrische Rehabilitation & Bereichsleitung Kurzzeitpflege	Modellklinik 2	MaPfD<br>BlD
AOK	25.07.2022	Mitarbeiter*in Competence Center Rehabilitation<br>Mitarbeiter*in Competence Center Rehabilitation	CC Reha AOK	Ma1cc<br>Ma2cc
